# Multiple sclerosis disease-modifying drug use by immigrants: a real-world study

**DOI:** 10.1038/s41598-023-46313-7

**Published:** 2023-12-01

**Authors:** Jonas Graf, Huah Shin Ng, Feng Zhu, Yinshan Zhao, José M. A. Wijnands, Charity Evans, John D. Fisk, Ruth Ann Marrie, Helen Tremlett

**Affiliations:** 1 Division of Neurology, Department of Medicine, Djavad Mowafaghian Centre for Brain Health, University of British Columbia, Vancouver, BC Canada; 2 Flinders Health and Medical Research Institute, College of Medicine and Public Health, Flinders University, Bedford Park, SA Australia; 3 College of Pharmacy and Nutrition, University of Saskatchewan, Saskatoon, SK Canada; 4 Departments of Psychiatry, Psychology and Neuroscience, and Medicine, Nova Scotia Health Authority, Dalhousie University, Halifax, NS Canada; 5 Departments of Internal Medicine and Community Health Sciences, Rady Faculty of Health Sciences, Max Rady College of Medicine, University of Manitoba, Winnipeg, MB Canada; 6 Division of Neurology, Department of Medicine, Djavad Mowafaghian Centre for Brain Health, University of British Columbia Vancouver, Rm S126, 2211 Wesbrook Mall, Vancouver, BC V6T 2B5 Canada

**Keywords:** Health services, Multiple sclerosis

## Abstract

Little is known about disease-modifying drug (DMD) initiation by immigrants with multiple sclerosis (MS) in countries with universal health coverage. We assessed the association between immigration status and DMD use within 5-years after the first MS-related healthcare encounter. Using health administrative data, we identified MS cases in British Columbia (BC), Canada. The index date was the first MS-related healthcare encounter (MS/demyelinating disease-related diagnosis or DMD prescription filled), and ranged from 01/January/1996 to 31/December/2012. Those included were ≥ 18 years old, BC residents for ≥ 1-year pre- and ≥ 5-years post-index date. Persons becoming permanent residents 1985–2012 were defined as immigrants, all others were long-term residents. The association between immigration status and any DMD prescription filled within 5-years post-index date (with the latest study end date being 31/December/2017) was assessed using logistic regression, reported as adjusted odds ratios (aORs) with 95% confidence intervals (CIs). We identified 8762 MS cases (522 were immigrants). Among immigrants of lower SES, odds of filling any DMD prescription were reduced, whereas they did not differ between immigrants and long-term residents across SES quintiles (aOR 0.96; 95%CI 0.78–1.19). Overall use (odds) of a first DMD within 5 years after the first MS-related encounter was associated with immigration status.

## Introduction

Canada’s long history of immigration, combined with its universal health care coverage and high prevalence of multiple sclerosis (MS) makes it an ideal region to study health service use in immigrants with MS. According to Statistics Canada’s Census data, in 2021 over one in five of the population (23%) were, or had ever been, a landed immigrant or permanent resident^1^. In addition, most (> 50%) of Canada’s recent immigrants entered under the economic category, while over one-third (35%) came via one of the skilled worker programs. While historically, the majority of immigrants came from Europe, this is no longer true, with most now coming from Asia, including the Middle East (62%), with 19% of all immigrants born in India and 10% from Europe. Nonetheless, the most (93%) recent immigrants could conduct a conversation in either of Canada’s official languages (English or French)^1^.

MS is a life-altering, chronic immune-mediated disease resulting in lesions and neurodegeneration in the brain and spinal cord. MS typically first presents in young to middle-aged adults, with most diagnosed between the ages of 20–50 years, and is the most common cause of non-traumatic neurologic disability in young adults^2^. Although there is no known cure for MS, it is now a treatable disease, with over 20 different disease-modifying drugs (DMD) approved for use. However, despite the potential barriers to health care which often disproportionately affect immigrants, including cultural and financial^3^, much remains unknown regarding use of these DMDs by immigrants with MS in countries, such as Canada, with universal health coverage. Prior work from Ontario, Canada found that, as compared to long-term residents, immigrants with MS had a lower socioeconomic status (SES) and also differed in terms of health care use. For example, while the rates of outpatient neurology visits were somewhat lower in the year before MS diagnosis (adjusted rate ratio 0.93; 95% confidence interval [CI] 0.87–0.99), rates were higher in the year during and after MS diagnosis (adjusted rate ratios 1.17; 95% CI 1.12–1.23 and 1.16; 95% CI 1.10–1.23, respectively)^4^. Hospitalization rates were higher during the year of diagnosis, but lower post-diagnosis. However, whether access to a DMD was affected could not be examined because population-based prescription drug data are not available for persons under age 65 years.

A recent call-to-action highlighted the need for better understanding of potential health disparities among immigrants, and the role of social determinants of health and consequent inequities in MS care that may underpin those disparities^5^. Key gaps in knowledge included the lack of fundamental epidemiological descriptions of healthcare use by immigrants, such as access to, and use of, one of the mainstays of MS management—the DMDs used to treat MS. Therefore, we assessed the relationship between immigration status and initiation of a DMD. We focused on the 5 years after a first MS-related healthcare encounter, in part because early [*versus* delayed] DMD treatment may result in better MS outcomes^6^. Further, we explored whether SES had a differential impact on DMD initiation between immigrants and long-term residents. We hypothesized that, despite universal health care coverage, DMD initiation would be lower in immigrants relative to long-term residents, and that it would be lowest for immigrants living in the most socioeconomically deprived neighborhoods.

## Results

**Figure 1:**
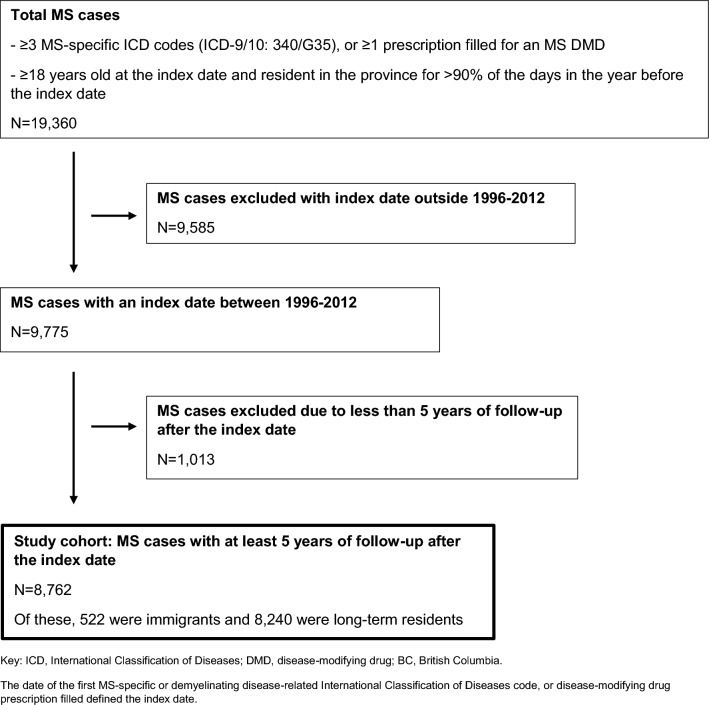
Flowchart of the multiple sclerosis (MS) cohort formation (1996–2017).

Overall, 8762 MS cases were identified (Fig. 1). Of these, 6% were immigrants. There was a similar female predominance for the immigrants and long-term residents, Table 1 (Pearson’s Chi Squared test p value 0.05, degree of freedom [df] 1).

**Table 1:** Characteristics of the multiple sclerosis cohort by immigration status (1996–2017).

Characteristics	Immigrants n = 522	Long-term residents n = 8240	*p* value*
Females, n (%)	368 (70.5)	6132 (74.4)	0.05
Age at the index date in years, mean (SD)	40.0 (11.2)	43.2 (12.4)	< 0.0001
Calendar period at the index date, n (%)			< 0.0001
1996–1999	64 (12.3)	2234 (27.1)	
2000–2004	121 (23.2)	2446 (29.7)	
2005–2009	210 (40.2)	2259 (27.4)	
2010–2012	127 (24.3)	1301 (15.8)	
Socioeconomic status^a^, n (%)			0.02
1 (lowest income quintile, most deprived)	120 (23.0)	1465 (17.8)	
2	86 (16.5)	1546 (18.7)	
3	115 (22.0)	1740 (21.1)	
4	94 (18.0)	1795 (21.8)	
5 (highest income quintile, most affluent)	103 (19.7)	1671 (20.3)	
Unavailable	< 6	23 (0.3)	
Comorbidity score^b^, n (%)			0.90
0	395 (75.7)	6316 (76.7)	
1	94 (18.0)	1379 (16.7)	
2	22 (4.2)	361 (4.4)	
≥ 3	11 (2.1)	184 (2.2)	
Filled a DMD prescription within 5 years of the index date, n (%)			0.90
Yes	138 (26.4)	1889 (22.9)	
No	384 (73.6)	6351 (77.1)	
By route of administration			
Injectable DMD	130/138 (94.2)	1826/1889 (96.7)	0.14
Oral or intravenous DMD	8/138 (5.8)	63/1889 (3.3)	
Persons immigrating to Canada as refugees, n (%)	68 (13.0)	N/A	
Region based on country of last permanent residence, n (%)		N/A	
Asia	215 (41.2)		
Europe	176 (33.7)		
United States of America	58 (11.1)		
Africa	30 (5.7)		
Latin America and the Caribbean	28 (5.4)		
Oceania	15 (2.9)		

By definition, the index date could also be the date of first DMD prescription filled. This occurred in a minority of cases (< 6/522 immigrants and 39/8240 [0.5%] long-term residents).

Between 1996 and 2017, the available DMDs comprised: the injectables (beta-interferon and glatiramer acetate); oral (fingolimod, dimethyl fumarate and teriflunomide), and intravenous (natalizumab). While daclizumab, alemtuzumab and ocrelizumab were available, these were never used as the first DMD within our multiple sclerosis population.

‘< 6’ indicates that the variable comprising less than 6 individual persons with MS were suppressed, as required by the data sharing agreement.

Follow-up was from index date until the earliest of: death; emigration from British Columbia; or study end (December 31st 2017).

*SD* standard deviation, *DMD* disease-modifying drug (used to treat MS), *N/A* not applicable.

^a^Socioeconomic status is represented by neighborhood income quintiles, based on the closest available measurement to the index date.

^b^Comorbidity is measured using the Charlson Comorbidity Index (modified to exclude hemiplegia/paraplegia to avoid misclassifying MS complications as comorbidity) during the 1-year period prior to the index date.

*Tests used include Pearson’s Chi-squared test (sex, calendar period, SES, comorbidity score, DMD prescription filled within 5 years), Fisher’s exact test for count data (DMD prescription filled by route of administration), and Wilcoxon rank sum test (age).

Most immigrants were resident in Asia (41%) or Europe (34%) before moving to Canada; 13% were refugees. A higher proportion of immigrants fell within the lowest (most deprived) SES quintile at the index date compared to long-term residents (23% *versus* 18%), Table 1 (Pearson’s Chi squared test p value 0.02, df 4). More than three-quarters of immigrants (76%) and long-term residents (77%) had no comorbidity at the index date, as measured using the Charlson Comorbidity Index, Table 1 (Pearson’s Chi squared test p value 0.90, df 3).

DMD use was captured within 5 years of the index date, thus the maximum relevant follow-up period was 1996–2009 for the index date group 1996–2004 and 2005–2017 for the index date group 2005–2012. More than one-quarter of immigrants (26%; 138/522) and 23% of long-term residents (1889/8240) filled a DMD prescription within 5 years after the index date, Table 1 (Fisher’s exact test p value 0.90). After adjusting for sex, age, comorbidity score, SES, and calendar years at the index date, the odds of filling a DMD prescription within 5 years after the index date were similar between immigrants and long-term residents with MS (aOR 0.96; 95% CI 0.78–1.19). In the analysis that included SES and immigration status, we observed a 17% decrease in the odds of filling a DMD prescription within 5 years after the index date for each decrease in SES quintile (representing a decrease in neighborhood-level income) for immigrants (aOR per SES-quintile decrease: 0.83; 95% CI 0.72–0.96) but not for long-term residents (aOR 0.99; 95% CI 0.95–1.03). Thus, at the lowest income quintile, the odds of filling a DMD became 32% (aOR 0.68; 95% CI 0.47–0.98) lower for the immigrants versus long-term residents (Table 2).

**Table 2:** Adjusted odds ratios of filling a disease-modifying drug prescription within 5 years after the index date for immigrants *versus* long-term residents in the multiple sclerosis population, by socioeconomic status (SES) quintile.

Socioeconomic status^a^	Adjusted odds ratio	95% confidence interval	β value	Standard error (SE)	β lower bound	β upper bound
1 (lowest income quintile, least affluent)	**0.68**	**0.47–0.98**	− 0.39	0.19	− 0.76	− 0.02
2	0.81	0.62–1.05	− 0.21	0.14	− 0.48	0.05
3	0.96	0.78–1.19	− 0.04	0.11	− 0.25	0.18
4	1.15	0.89–1.48	0.14	0.13	− 0.11	0.39
5 (highest income quintile, most affluent)	1.37	0.96–1.95	0.32	0.18	− 0.04	0.67

Derived from the complementary analysis where an interaction between SES and immigrant status was introduced.

^a^Socioeconomic status is represented by neighborhood income quintiles, based on the closest available measurement to the index date.

Bold indicates 95% confidence interval did not include 1.

*Post-hoc*, we described the mode of DMD administration for persons who had filled a DMD prescription within the 5-year period (Table 2). Most immigrants (94.2%) and long-term residents (96.7%) first used an injectable drug (beta-interferon, glatiramer acetate), Table 1 (Fisher’s exact test p value 0.14). Although there was a suggestion that immigrants were more likely to use an oral or intravenous DMD (5.8% of immigrants did so *versus* 3.3% of long-term residents), a higher proportion of immigrants (*versus* long-term residents) also had their index date in later years (2005–2012) when oral and intravenous DMDs were available.

## Discussion

In this population-based study, the overall use (odds) of a first DMD prescription fill within 5 years after the first MS-related healthcare encounter was similar between immigrants and long-term residents in BC, Canada. However, immigrants with a lower (*versus* higher) area-level SES, based on neighborhood-level income, had lower odds of filling a first DMD prescription, while this association was not seen in long-term residents. Therefore, our hypothesis was confirmed. This suggests that intersectionality of immigrant status and SES contributes to disparities in DMD access^5^. Whether this adversely affects outcomes for immigrants with MS warrants future study.

DMD use in immigrants has seldom been reported, and there were few studies with which to compare our findings. A largely descriptive, population-based study from France (1995–2007) which focused on DMD-treated individuals suggested that immigrants (n = 133, all of whom were from North Africa) were treated earlier with a DMD, as compared to 2288 age- and sex matched MS cases from across continental Europe (1.3 *versus* 4.5 years after MS diagnosis)^7^. A smaller study from Oslo, Norway observed that for persons diagnosed with MS after the first DMDs became available (i.e., 1997) a somewhat higher proportion (71%; 27/38) of non-Western immigrants (from the Middle East, Asia, Africa or South/Central America) compared to 59% (143/241) of Norwegian individuals used any DMD treatment over the 6 year study period (1997–2012)^8^. While differences in study design, and also the time periods studied, makes it rather difficult to compare across articles, reassuringly both these studies and ours suggest that overall, of the relatively few immigrants studied to date, differences in DMD initiation by immigration status have not been observed, at least at the broad level in regions with access to universal healthcare. However, our findings indicated that health disparities amongst immigrants were affected by SES; the odds of filling a first DMD prescription was nearly one-third (32%) lower for those in the lowest, i.e., least affluent, SES quintile, relative to long-term residents in that same quintile. Health disparities related to socioeconomic inequities have been well-recognized in the wider population and represent a significant public health challenge^9^. Even within regions, such as Canada, with universal healthcare coverage, health disparities persist in lower socioeconomic groups, indicating that equitable availability of healthcare services alone is insufficient to address such disparities comprehensively^9, 10^. Individuals, including immigrants from lower socioeconomic groups can face several barriers that impact their overall health, including, although not limited to, health literacy, transportation challenges, work obligations, and cultural beliefs^11–13^. Lower SES has been associated with a higher disease burden, including higher rates of physical and mental health morbidities, and higher mortality rates in both the MS and general populations^4, 9, 14–17^, with immigrants often disproportionately affected by comorbidities^18^. A deeper understanding of the underlying mechanisms driving these disparities in the MS population and impact on health outcomes could help inform evidence-based interventions for reducing health inequalities.

Our study has several limitations. Our immigration-related information was only available up until 2012, limiting our index date to 1996–2012. Further, our analysis lacked MS-specific clinical information such as relapses and disease severity. It would be of interest for future studies to consider the clinical status of immigrants in relation to DMD uptake. For example, prior work from Canada found that immigrants had a higher rate of hospitalizations in the year of their MS diagnosis versus long-term residents (adjusted rate ratio 1.20; 95% CI 1.04–1.39)^4^. This could suggest that immigrants were experiencing a more severe disease course even at presentation (diagnosis). Diagnostic delays in other under-represented or marginalized groups have been reported, including in MS^5^. This can lead to higher disability at the time of diagnosis, and possibly a greater need to treat rapidly with highly effective DMDs^5^. Whether immigrants have an actual altered risk of MS is less clear; a 2019 Canadian study found that MS incidence varied widely by region of origin, being higher for some regions, such as the Middle East, while lower for most others, as compared to immigrations from Western regions. MS incidence also varied by time spent resident in Canada^19^. Further work is warranted to explore all these issues further. Due to the cohort size and the lack of information on the duration of residency in the province for all members of the cohort, we were constrained in performing subgroup analyses (e.g., by age^20^ as ≤ 6 immigrants and ≤ 6 long-term residents aged ≥ 55 years at the index date had ever filled a DMD prescription). Strengths of our study included the use of linked health administrative data, and the large, geographically-defined MS population identified within a region providing universal healthcare, which included access to the DMDs to treat MS. Moreover, the mandatory enrollment in the publicly available health system virtually eliminates volunteer or selection bias and the prospective collection of the data ensure that our findings are not affected by recall bias.

To conclude, the use (odds) of any DMD to treat MS within 5 years after a first MS-related healthcare encounter differed between immigrants and long-term residents in a universal health care setting: SES affected findings, suggesting that immigrants (but not long-term residents) living in lower (*versus* higher) income neighborhoods may face health disparities, at least when it comes to prompt use of a DMD to treat MS. How this affects longer term outcomes for immigrants with MS, and what factors may drive potential health disparities for these individuals, requires consideration.

## Methods

### Data sources and cohort creation

We performed a population-based study in the province of British Columbia (BC), Canada using linked health administrative data. BC has a public healthcare plan, with mandatory enrollment for residents. Encounters with the healthcare system are routinely collected, including physician and hospital visits and prescription drugs dispensed in the community or outpatient setting. Across Canada, over 98% of the population (comprising both immigrants and long-term residents) have access to medically necessary services without user fees, including consultations with specialists or general physicians for the treatment of chronic diseases such as MS^21^. Data access was facilitated by Population Data BC and databases used included: Medical Service Plan Billing Information (providing physician visits [‘claims’])^22^ and the Discharge Abstract Database (providing hospital admissions/discharges)^23^. Both provided diagnoses made by physicians, coded using the International Classification of Diseases (ICD) system. PharmaNet provided information on prescriptions filled at outpatient/community pharmacies, including dates and unique drug identification numbers^24^. Census Geodata provided SES estimates based on the median neighborhood-level income. Neighborhood income was derived by linking the individual’s postal code to the census dissemination area, using a Statistics Canada algorithm^25^. Registration and Premium Billing files provided demographics (sex, date of birth, and place of residency via the first 3-digits of each person’s postal code) and confirmation of residency in BC via days registered in the mandatory healthcare plan^26^. Vital Statistics captured death dates^27^. Finally, the Immigration, Refugees and Citizenship Canada Permanent Residents dataset^28^ captured all landed permanent residents in Canada (1985–2012) and included refugee status and country of last permanent residence.

We identified MS cases using a validated algorithm requiring the presence of at least three MS-specific ICD codes (ICD-9 340 and ICD-10-CA G35) related to a hospitalization or physician visit in any combination, or at least one prescription filled for an MS DMD (Table 3)^29^. For example, an individual could fulfill the algorithm if they had three ICD-9 340 codes, or two ICD-9 340 and one ICD-10-CA G35 codes, and so on. The date of the first MS-related healthcare encounter defined the index date. Tables 3 and 4 show all relevant ICD codes and DMDs. MS cases were eligible for inclusion if they were: ≥ 18 years old at the index date and resident in BC for > 90% of the days in the year pre-index date^30^. The earliest possible index dates were 1-January-1996 and latest were 31-December-2012. Both dates reflect data availability; prescription data started 1-January-1996 and the immigration data ended 31-December-2012. Also, 1996 was the first full calendar year that the DMDs became available to treat MS in Canada. MS cases were categorized as an immigrant or long-term resident. Immigrants were defined as persons with a record of being a landed permanent resident at any time between 1985 and 2012. All others were considered long-term residents (i.e., persons who had received landed permanent resident status before 1985 or who had always resided in Canada). All persons were followed from their index date until the earliest of emigration from BC (i.e., end of mandatory healthcare plan registration), death or study end (31-December-2017). At least 5 years of follow-up (inclusive of the index date) were required in order to examine the study outcome—any DMD prescription filled within the five years post-index date. This five year period represented ‘early’ initiation of a DMD which is thought to result in better MS outcomes^6^. Five years also represented the time between the latest possible index date (31-December-2012) and the latest study end (31-December-2017), thus maximizing the number of eligible persons.

**Table 3:** The disease-modifying therapies approved by Health Canada to treat multiple sclerosis (1995/6–2017): drug name (brand/generic), drug identification number, mode of administration (injectable, oral, intravenous), Health Canada approval date.

Disease-modifying therapy	Drug identification number	Mode of administration	Health Canada approval date
Betaseron® (IFNB-1b)	02169649	Injectable	July 1995
Extavia® (IFNB-1b)	02337819	Injectable	November 2009
Avonex® (IFNB-1a)	02237770	Injectable	April 1998
	02269201		
	02267594		
Rebif® (IFNB-1a)	02281708	Injectable	February 1998
	02277492		
	02237317		
	02237319		
	02237320		
	02277492		
	02281708		
	02318253		
	02318261		
	02318288		
Plegridy® (Peg-IFNB-1a)	02444372	Injectable	August 2015
	02444380		
	02444399		
	02444402		
Copaxone® (glatiramer acetate)	02233014	Injectable	October 1997
	02245619		
	02456915		
	02441446		
	02481510		
Glatect® (glatiramer acetate)	02460661	Injectable	August 2017
Tysabri® (natalizumab)	02286386	Intravenous	September 2006
Gilenya® (fingolimod)	02365480	Oral	March 2011
	02482533		
Tecfidera® (dimethyl fumarate)	02404508	Oral	April 2013
	02420201		
Aubagio® (teriflunomide)	02416328	Oral	November 2013
Lemtrada® (alemtuzumab)	02418320	Intravenous	December 2013
Zinbryta® (daclizumab)	02459620	Injectable	December 2016
	02459639		
Ocrevus® (ocrelizumab)	02467224	Intravenous	August 2017

The DMDs listed represented all those available (approved) for use in MS by Health Canada at some point during the study. Daclizumab was withdrawn from the market in March 2018 due to safety concerns.

**Table 4:** Multiple sclerosis-specific and demyelinating disease related codes.

	ICD-9	ICD-10-CA
Multiple sclerosis-specific		
Multiple sclerosis	340	G35
Demyelinating disease-related		
Optic neuritis	377.3	H46
Acute transverse myelitis	323.82	G37.3
	341.2	
Acute disseminated encephalomyelitis	323	G36.9
Demyelinating disease of CNS unspecified	341.9	G37.8
Other acute disseminated demyelination	Not applicable	G36
Neuromyelitis optica	341.0	G36.0

*ICD-9-CM* International Classification of Diseases, Ninth Revision, Clinical Modification, *ICD-10-CA* International Classification of Diseases, Tenth Revision, Canada.

The outcome of any DMD prescription filled in the 5-years post-index date was categorized as ‘yes’ or ‘no’. Over the study period (1996–2017), the available DMDs comprised: the injectable (beta-interferon and glatiramer acetate); oral (fingolimod, dimethyl fumarate and teriflunomide), and intravenous (natalizumab) drugs. While daclizumab, alemtuzumab and ocrelizumab were available, these were never used as the first DMD within our MS population. Further details, including brand names and DMD identification numbers are in Table 3.

### Statistical analysis

We described characteristics at the index date according to immigration status including sex, age, calendar years (i.e., 1996–1999, 2000–2004, 2005–2009, 2010–2012), SES (quintiles ranging from 1 [lowest income quintile, i.e., most deprived] to 5 [highest income quintile, i.e., most affluent]), comorbidity score (0, 1, 2, and ≥ 3) measured using the modified Charlson Comorbidity Index^31^ (based on physician/hospital data during the 1-year period pre-index date), and the number of persons filling a DMD within 5 years after the index date. For immigrants only, we described their refugee status and place of last permanent residence (grouped by region: Asia, Europe, United States of America, Africa, Latin America and the Caribbean, and Oceania). We assessed the relationship between immigration status and filling a DMD prescription within 5 years post-index date using a logistic regression model, adjusted for sex, and, at the index date: age (continuous), comorbidity score, SES, and calendar years (see above). Findings were reported as adjusted odds ratios (aOR) with 95% CIs. Pearson’s Chi-squared test (sex, calendar period, SES, comorbidity score, DMD prescription filled within 5 years), Fisher’s exact test for count data (DMD prescription filled by route of administration), and Wilcoxon rank sum test (age) were used to assess cohort characteristics (Table 1).

In order to analyse whether SES had a differential effect on DMD initiation between immigrants and long-term residents, we further included an interaction between SES and immigration status. We used a similar approach as in the main analyses, but with the quintiles as a continuous measure, and after excluding the minority (< 0.4%) of MS cases without an available SES.

### Ethics, registration, and patient consents

This study was part of a wider research program registered with ClinicalTrials.gov (NCT04472975), and ethical approval was obtained from the Research Ethics Boards at the University of British Columbia (#H18-00407; waiver for consent granted).

## Data Availability

As we are not the data custodians, we are not authorized to make the data available. With the appropriate approvals, the data may be accessed through the Population Data British Columbia.
